# Psychiatric Genetics, Epigenetics, and Cellular Models in Coming Years

**DOI:** 10.20900/jpbs.20190012

**Published:** 2019-08-22

**Authors:** Chunyu Liu, Stephen V. Faraone, Stephen J. Glatt

**Affiliations:** Department of Psychiatry, SUNY Upstate Medical University, Syracuse, NY 13210, USA

**Keywords:** psychiatry, genetics, epigenetics, behavior, population diversity, function study, drug response, cellular phenotype, molecular phenotype, regulatory network

## Abstract

Psychiatric genetic studies have uncovered hundreds of loci associated with various psychiatric disorders. We take the opportunity to review achievements in the past and provide our view of what is coming in the fields of molecular genetics, epigenetics, and cellular models. We expect that SNP-array and sequencing-based studies of genetic associations will continue to expand, covering more disorders, drug responses, phenotypes, and diverse populations. Epigenetic studies of psychiatric disorders will be another promising field with the growing recognition that environmental factors impact the risk for psychiatric disorders by modulating epigenetic factors. Functional studies of genetic findings will be needed in cellular models to provide important connections between genetic and epigenetic variants and biological phenotypes.

## INTRODUCTION

Molecular genetic research into psychiatric disorders has made substantial progress in the last several decades, delivering important findings about the genetic risks of several conditions. Fast-evolving genotyping and sequencing technologies have driven this progress. The most important findings came from genome-wide association studies (GWASs), whole-exome sequencing (WES), and whole-genome sequencing (WGS) of various disorders, behavioral traits, and other phenotypes. GWASs has produced the majority of findings in the past ten years [1]. We have learned that common single nucleotide polymorphisms (SNPs), rare copy number variants (CNVs), and rare *de novo* mutations all contribute substantially to the etiology of psychiatric disorders like schizophrenia (SCZ), bipolar disorder (BD), autism spectrum disorder (ASD), attention-deficit/hyperactivity disorder (ADHD) and others. However, these variants still account for only a small fraction of all the genetic risk for these highly heterogeneous disorders, and much of the heritability remains to be explained. Moreover, the biological significance of those disease-associated variants and the true causal factors remain to be discovered.

Which major research areas of psychiatric genetics will yield major breakthroughs in the coming years? Research on deep phenotypes, new phenotypes, epigenetics of large cohorts, cross-disorder analyses, pharmacogenetics, and cellular models will likely bring novel understandings about the etiology and pathology of psychiatric disorders, and better guide development of new treatments. Herein we review a few approaches, including genetics, epigenetics, and molecular and cellular functional studies, and preview their anticipated impacts on the field.

## GWAS/WES/WGS WILL CONTINUE TO EXPAND, COVERING MORE DISORDERS, DRUG RESPONSES, PHENOTYPES, AND DIVERSE POPULATIONS

GWASs will continue to grow in terms of sample size for major psychiatric disorders and will expand to include other heritable disorders. More importantly, researchers designing GWASs should pay attention to population diversity and include under-studied ancestral groups, especially those groups that will help narrow blocks of linkage disequilibrium in the search for causal variants. Pharmacogenetics must catch up with disease GWAS. Gradually, phenotyping will steal center stage from genotyping, but the change will be gradual as phenotyping is more complex and more expensive.

The Psychiatric Genomics Consortium (PGC) currently comprises large-sample studies of SCZ, BD, ADHD, major depression, Tourette Syndrome, obsessive-compulsive disorder, post-traumatic stress disorder, ASD, eating disorder, anxiety, substance use disorder, and Alzheimer’s disease. Those studies will continue to grow, leading to discoveries of more common-SNP associations with smaller effects, and polygenic risk scores (PRS) capable of explaining greater variance with increased precision. Several common psychiatric disorders with clear genetic risks have received minimal genetic research efforts to date, including personality disorders [2], sleep disorders [3], conduct disorder [4] and learning disabilities [5].

The population diversity of psychiatric GWASs has been relatively limited to date. The majority of GWASs included only samples from patients of European ancestry. A smaller number of studies involved Asian patients (Han Chinese [6–15], Japanese [16–19], Korean [20], Indian [21,22], Pakistani [23]). Even fewer studies have been completed on populations originating from Africa or South America. From the researcher’s point of view, the inclusion of diverse populations in genetic studies can improve the resolution of genetic maps and enhance our ability to identify specific risk genes and regulatory elements in the human genome. In terms of eliminating health disparities, the inclusion of diverse populations in genetic studies is the only way for all humanity to benefit from modern genetics research.

Pharmacogenetics focuses on identifying genetic factors that influence treatment response, including efficacy and side effects. Although its clinical significance is obvious, pharmacogenetics research into psychiatric diseases is in its early stages, having fewer than 5000 samples tested to date per drug studied. GWASs have been performed on the therapeutic effects and side effects of antipsychotics, selective serotonin reuptake inhibitors, and lithium. As we observed, however, GWASs of drug response to date did not recover any of the top pharmacodynamic candidate genes, such as CYP2D6 and CYP2C19. Future, sufficiently powered GWASs investigating the efficacy and side effects of treatment should better predict appropriate drug choices than current candidate genes. Relating individual genes, gene sets, or polygenic scores to treatment response is the foundation for developing precision medicine. Pharmacogenetic GWASs are more expensive than disease GWASs due to the complexities of the clinical setting, their demand of time and personnel investment for collecting treatment-related data, and the greater degree of patient involvement. The PGC’s organizational model of combining data from many small studies could be one of the solutions for building powerful pharmacogenetics datasets. Acquiring data from major medical institutes that have large medical record data collections coupled with biobanks should also be pursued. Meanwhile, current data from small studies are being compiled and interpreted for clinical use by the Clinical Pharmacogenetics Implementation Consortium (CPIC; https://cpicpgx.org/). Researchers should also be aware of an ongoing debate about best practices in establishing the clinical utility of pharmacogenetic findings [24].

Intermediate phenotypes (also called endophenotypes) are quantitative biological traits that are believed to mediate the effects of genes on disorders while having a simpler genetic architecture than the disorders themselves. Such phenotypes have attracted researchers for two major reasons: (1) they can be used to construct a causal framework of complex traits leading to disorders; therefore, they may help to elucidate the biological path between genetic variants and, ultimately, higher-order disease phenotypes; and, (2) they could have a more direct connection to underlying genetics than to the disorder *per se*. GWASs of intermediate phenotypes could be more powerful than disease GWASs if the simpler genetic architecture of the former is, as hypothesized, associated with larger effect sizes for GWAS SNPs. Consequently, the genetics of intermediate phenotypes could be used to explain GWAS signals (via, for example, Mendelian randomization studies) or to facilitate the identification of disease risk genes.

Several projects have pioneered the collection of intermediate phenotype data from large cohorts. Within the NIMH Research Domain Criteria (RDoC) initiative, the Bipolar and Schizophrenia Network for Intermediate Phenotypes (BSNIP) project has collected deep phenotype data, including cognitive functions, brain imaging, brain electroencephalogram, and eye movement, in patients with BD and SCZ. The Philadelphia Neurodevelopmental Cohort [25] has intensive data of cognitive functions. The SUNY Upstate Reward Regulation Project (SUREREG) is studying the genetics of the RDoC Positive Valence System, which comprises several reward-related phenotypes. Brain imaging data has been the focus of the ENIGMA consortium [26].

Several other population- or community-based projects, including the UK Biobank, the Million Veteran Program (MVP), the National Institute of Health (NIH)’s “All of Us”, Adolescent Brain Cognitive Development (ABCD) and 23andMe, should yield important insights into the genetics of many behavioral traits, such as personality, sleep, education, smoking, brain volume and brain connectivity, plus a host of cognitive traits. These big data projects have extensive phenotype data originating from numerous fields. They will offer unique opportunities to study phenotypic and genetic relationships among various disorders and traits. Some of the relationships will be causal, while others will not. Pleiotropic effects will be a common confounder in isolating causal effects. Resolving their relationships will be informative in revealing disease mechanisms.

Developing biobanks coupled with electronic health records (EHR) is an attractive approach to establish large datasets that can be used for studying the biology of various diseases, including psychiatric disorders. Vanderbilt University, Partners HealthCare, and the Mayo Clinic are a few institutes leading the efforts to implement hospital-wide programs. BioVU (https://victr.vumc.org/pub/biovu/) is the Vanderbilt biobank contains medical records of more than 200,000 subjects. The Partners HealthCare Biobank (https://biobank.partners.org/) serves affiliated hospitals in the Boston area. More than 100,000 subjects have been collected by them so far. About 30,000 subjects have been collected by the Mayo Clinic Biobank. Moreover, Mayo Clinic has a separate Biobank specific for bipolar disorder [27]. The Electronic Medical Records and Genomics (eMERGE) network [28], involving ten institutes including Vanderbilt, MGH, and Mayo clinic, has been the major NIH-funded project utilizing medical records for genomic research. ADHD and ASD are part of the project. Otherwise, current EHR-biobank projects do not have a major emphasis on psychiatric disorders yet.

Besides those commonly studied phenotypes described above, we should also adopt and develop new phenotypes that can be measured more efficiently within populations using mobile devices and that have the underlying genetics of large effect size. Mobile device-based digital phenotyping is currently a hot topic with great potential for many creative uses in genetics. Digital phenotyping enables fast collection of large data sets [29,30]. New phenotypes can target traits of potentially large genetic effect size. These are two different approaches that would boost statistical power to detect the genetics of new phenotypes. The discovered SNP-associated phenotypes can be used to annotate functions of the SNPs. The phenotype-annotated SNPs can be used to refine the study of disorders. Algorithms like PrediXcan [31] and MetaXcan [32](or sPrediXcan) and MulTiXcan [33] will benefit from these data to perform gene-based analyses. On the other hand, genes and pathways associated with intermediate phenotypes will help to dissect mechanism, explain the biology of psychiatric disorders, and many other traits through the technique of phenome-wide association study (PheWAS)[34].

Not all phenotypes are equal in terms of the effect sizes of their genetic factors. Good examples of phenotypes with large effect sizes are molecular phenotypes. Molecular phenotypes, like gene expression and DNA methylation have triumphed in the mapping of regulatory elements. One hundred samples are sufficient to map expression and methylation quantitative traits (eQTLs and mQTLs)[35,36]. Inflammation is another highly interesting phenotype that deserves genetic mapping, given its potential involvement across many psychiatric disorders; nevertheless, only limited studies have been performed so far. One GWAS study of circulating cytokines and growth factors pioneered the mapping of 27 loci [37]. Cellular studies will provide additional promising new phenotypes that will be discussed later in this essay.

Since many more samples will be studied with many more phenotypes including common variants of weak-effect and rare variants of relatively strong effect, these will implicate risk genes and regulatory variants for disease onset, persistence, and treatment response. Polygenic risk scores for traits and disorders will be further refined. But genetics alone will not be enough to accomplish the goals of better disease classification, diagnosis, and treatment; the power of epigenetics and functional studies will be needed to improve the resolution and help uncover mechanisms. And, to fully understand the etiology of psychiatric disorders, the interaction of environmental and genetic risks will require clarification.

Cross-disorder studies will be particularly fruitful in the coming years for psychiatry. It is expected that most psychiatric disorders share some genetic risk variants. The degree of sharing likely defines the degree of shared clinical features among disorders. Cross-disorder studies will not only identify risk genes that contribute to multiple disorders but will also help to identify genes and other genomic features that may differentiate disorders. Cross-disorder genetic and epigenetic studies may eventually redefine current disease classifications, becoming biology-based, objective classifications.

## EPIGENETICS OF PSYCHIATRIC DISORDERS WILL BE PURSUED, PROMPTED BY THE EVIDENCE THAT ENVIRONMENTAL FACTORS CHANGE EPIGENETICS AND RISK FOR PSYCHIATRIC DISORDERS

Epigenetic factors, including DNA methylation, histone modifications, non-coding RNAs, and others, are dynamic regulators of gene expression. They are frequently products of interactions between genetic and environmental factors. Epigenetics is a critical part of the formula to classify disorders and predict risks and treatment response. Normally, epigenetic profiling will be performed along with transcriptome profiling, since the chief functional impact of epigenetic modification is thought to alter gene expression.

The epigenotype is the signature of epigenetic marks, the equivalent of DNA genotype. Profiling epigenotypes is more expensive than genotyping and is fraught with technical uncertainties. DNA genotypes are typically biallelic; whereas epigenotypes are mostly quantitative, and their measurement is frequently unstable. Influenced not only by many technical and biological factors, but epigenotypes are also regulated by genetic and environmental factors. DNA methylation is currently the most accessible epigenotype. It costs several hundred to three thousand dollars to assay one sample of one of two types of DNA methylation (5mC and 5hmC)[38], both of which may represent distinct regulatory functions. 5hmC and non-CpG methylation may carry important regulatory information or case-control differences that have been overlooked in the past because of the limitation of technologies used for profiling.

An epigenome-wide investigation into psychiatric disorders or treatment response is early in development. Longitudinal studies and large consortium projects will be needed to fulfill its promise. Unlike genetics, epigenetics must consider tissue-specificity, age, sex, treatment status, environmental exposure, and other factors. Longitudinal studies of peripheral tissues could track the epigenetic changes associated with different clinical symptoms and treatment stages while serving as a source of useful biomarkers. Unfortunately, most epigenetic studies today are still cross-sectional or case-controlled. Few have been published with two or more time points. The cost of collecting such data is the major limiting factor, as epigenotyping and following-up with patients to collect biospecimens are prohibitively expensive. Instead, a large biobank or collaborative consortium may be necessary to accelerate research progress. Small studies have insufficient statistical power, despite their superior quantitative characteristics of continuous epigenetic data relative to dichotomous genotype data. For this reason, small studies should be designed to follow a consensus design, using standard quality controls to readily combine small data into large analyses.

Gene expression and epigenetic profiles are specified by tissue and cell type. While tissue or cell-type specificity is a big challenge, it also presents an opportunity. As in transcriptome studies, profiling epigenetics in mixed cells could mask differences in subgroups of cells. Single-cell assays are gradually moving to the center stage of research. Nonetheless, applying single-cell assays to large populations still faces a cost barrier. Creative computational and statistical methods could be used to deconvolute the data from mixed cells or gross tissues [39–42], although some details could be lost in deconvolution. Moreover, the validity of those deconvolution methods remains to be proven. The compromise between resolution and comprehension in single-cell assays must be balanced, contingent upon specific hypothesis.

Epigenetic markers are not only regulators of gene expression and mediators of high-level phenotypes, including disease diagnoses or behavioral phenotypes; they are also products of genetic-environmental interactions. Medication, drinking, smoking, psychological stress, diet, and many other environmental conditions can provoke epigenetic changes. Integrating epigenetics with genetics and with other –omics may help to resolve some causal contributors to psychiatric disorders.

Environmental factors are known to contribute to epigenetic changes and disease risks. Several prenatal and postnatal factors, associated with alterations of DNA methylation in rodent model experiments and in human patients, may ultimately contribute to disease risk [38]. Alcohol and smoking are the two most studied factors [43–47]. Sex is another very important factor associated with both differential DNA methylation and differential risks for psychiatric disorders [48]. Many environmental factors promote neuroinflammation [49–51], increase oxidative stress [52,53], disturb neuronal development [54,55], and disrupt neuronal networks [55,56]. Such mechanistic models, connecting environmental factors as well as epigenetic and genetic variations to disease risks could be studied in large human populations, including both healthy and affected participants.

Relating peripheral biomarkers to changes in the central nervous system could employ information gleaned from *postmortem* brains. The PsychENCODE project [57] concentrates a rich resource of brain -omics data for psychiatric studies. It has the advantage of both depth and breadth by collecting multiple types of data from the same individuals while profiling a large number of individuals. PsychENCODE is one of the largest datasets of postmor*t*em brain samples from patients with SCZ, BD, and ASD. After the first set of publications [58–67], the data will be made available to the public for further mining. Much more remains to be discovered from these data.

## FUNCTIONAL FOLLOW-UP OF GWAS RESULTS WILL BE PERFORMED IN CELLULAR MODELS

Identifying GWAS associations is only the first critical step in understanding how a genetic variant can affect the risk for a complex disease. Fine-mapping of associated loci is essential to determine which of several variants in a haplotype block may be truly responsible for the association signal. But identifying causal variation also falls short of having a practical impact, as simply knowing the identity of the variant does not suggest a clinically actionable route, neither a specific gene nor a biological pathway. Thus, the mechanistic study of associated loci is the necessary next step. GWASs alone have limited values without functional follow-up studies.

Functional follow-up studies of GWAS “hits” (regions of the genome associated with the disease in question) have three primary goals: (1) identifying target genes of disease-associated variants; (2) establishing regulatory relationships, pathways, or networks among all associated variants and genes; and (3) revealing functional impacts of the associated genetic variants and genes on phenotypes at all levels, from molecular and cellular phenotypes to behavioral traits and disorders. These studies pose the greatest challenge for future research. We expect that shortly, cellular models will be the most productive field for discovering target genes, gene networks, and cellular functions relevant to psychiatric illness.

The biological significance of most GWAS signals remains elusive, without even a clear target gene or regulatory sequence for many associated SNPs. Given that an associated SNP could impact the expression of a distant gene, functional studies should begin with those associated SNPs. Brain eQTL and Hi-C data can be used to connect SNPs to their putative target genes. Reporter gene assays, knockdown and overexpression, and CRISPR (clustered regularly interspaced short palindromic repeats) editing can be used to validate the functional impact of specific SNPs and the putative regulatory elements where the SNPs reside [68–71]. Most published research to date typically concludes with an SNP regulating gene expression. Few studies investigate cellular phenotypes, which should be an important focus in the coming years.

Data integration and network construction will generate new knowledge of biological systems, including causal factors, underlying disease risks, and drug responses. Since genetics, multi–omics, and dimensional phenotype data will be generated from large populations, sorting out their relationships and causal connections will be important to resolving the puzzle of disease biology. Computational methods like weighted gene coexpression network analysis (WGCNA) and others have been used to build correlation-based networks. Construction of regulatory networks is a fast-evolving field. Networks can be developed based on quantitative correlations, physical interactions, and literature of biochemical experiments. High-throughput methods will speed up the growth of knowledge about networks. It should be noted that regulatory relationships predicted by *in silico* analyses need to be experimentally validated. Cellular experiments similar to those done for POU3F2 [59] and DGCR5 [58] will help to construct regulatory networks of gene expression.

Cellular phenotypes are phenotypes that can be measured in cells within a culture or within the living body. Commonly accessible cellular phenotypes include cell morphology, cell functionality, and cell division and differentiation speed, ion channel properties, electrophysiology, cellular responses to drugs or other insults, and interaction with other cells. Cellular phenotypes are another level of intermediate phenotypes that represent functional effects. They reflect biological changes associated with disease risk or with drug response. Cellular phenotypes bridge molecular phenotypes and high-level traits of behavior, brain structure, and function. This field requires tremendous investment to reduce cost and increase throughput. The challenges and potential solutions for using cellular models to study psychiatric disorders have been reviewed [72–74]. Molecular profiling coupled with high-throughput techniques that measure cellular phenotypes *in vivo*, *ex vivo*, and at the single-cell level, will advance the field significantly.

Identifying cellular phenotypes of genetic variants is different from uncovering a complete molecular mechanism, which requires detailed characterization of each molecule and its physical and biochemical interactions in the process—from the genetic variants to the phenotypes, like electrophysiological measures. Normally, establishing the complete biological mechanism demands extensive experimental steps, including the screening and manipulation of relevant genes and environmental conditions. In contrast, identifying cellular phenotypes associated with genetic variants of both direct and indirect causal relationships would be a reasonable alternative, associating a given cellular phenotype to one gene or genetic variant even while intermediate factors remain unknown. Cellular phenotyping is a readily practical approach for advancing large-scale screening, an essential component of genetic studies. Certainly, establishing valid cellular phenotypes and developing high-throughput phenotyping methods still require major investment.

## A REMAINING CHALLENGE: GENE NETWORKS AND THE OMNIGENIC MODEL

Current GWASs clearly show that psychiatric disorders involve hundreds, even thousands, of genes. The omnigenic model even proposed that almost every gene could contribute to disease risks and that “peripheral genes” cumulatively contribute more heritability through trans-effects than “core genes” do through cis-effects [75,76]. Although the omnigenic model is still actively debated, it offers a new perspective about the genetics of complex disorders [77–85]. For the time-being, it is hard to appreciate the complete biological meaning of genetic findings for all psychiatric disorders. Although only a few specific genes, such as C4, CACNA1C, DRD2, and a few others are the primary focus of many hypotheses, they account for a tiny fraction of disease genetic associations. The same is true for polygenic risk scores and top GWAS signals. We are still “blind men feeling the elephant.” Instead, the solution may reside in understanding how proteins coded by implicated genes interact with one another and with regulatory variations identified with GWASs. Recognizing these coded proteins will, in turn, help us to understand their related upstream and downstream biology. Genetic and epigenetic studies, along with cellular experiments, will piece together these networks and improve our understanding of the biological systems underlying psychiatric disorders.

## SUMMARY AND CONCLUSIONS

In the 1930s, researchers discovered that genetics contribute to psychiatric disorders [86]. Since then, particularly in the last two decades, psychiatric genetics has evolved into an extensive research field and has achieved significant progress. Advanced new technologies and statistical methods made genetic and epigenetic exploration possible, developing more phenotypes in larger populations and in different biological levels. In another ten to twenty years, we will not only have a better understanding of the risk for psychiatric disorders, but we should also have a better understanding of drug-target options for treating them.

Major data resources and consortium projects discussed in this paper are summarized in Table 1.

## Figures and Tables

**Table 1.** Major biobanks and consortium projects for psychiatric genetics and epigenetics.

Biobanks/Projects	Data	Websites
*Psychiatry-specific*		
Psychiatric Genomics Consortium	GWAS of major psychiatric disorders	https://www.med.unc.edu/pgc/
PsychENCODE	Epigenomics of postmortem brains	https://www.nimhgenetics.org/resources/psychencode
Philadelphia Neurodevelopmental Cohort	Deep phenotyping project	https://www.med.upenn.edu/bbl/philadelphianeurodevelopmentalcohort.html
Mayo Clinic Bipolar disorder biobank	Bipolar disorder biobank	https://www.mayo.edu/research/centers-programs/bipolar-disorder-biobank
BSNIP	Deep phenotyping project	http://b-snip.org/
ConLiGen	Consortium on Lithium genetics	http://www.conligen.org/
ENIGMA	Consortium on neuro-imaging genetics	http://enigma.ini.usc.edu/
*General*		
GTEx	Genetics of gene expression and regulation	https://gtexportal.org/home/
UK Biobank	Genetics of diseases and traits of 500k UK people	https://www.ukbiobank.ac.uk/
MVP	Genetics of diseases and traits in US veteran	https://www.research.va.gov/mvp/
All of Us	Research program on environment, life style and biology of 1 million people	https://allofus.nih.gov/
ABCD	Adolescent Brain Cognitive Development Study, the largest long-term study of brain	https://abcdstudy.org/
BioVU	Vanderbilt’s biorepository linked with EHR	https://victr.vumc.org/biovu-description/
Emerge	EHR-Biobank consortium	https://emerge.mc.vanderbilt.edu/
